# New Clinical Decision Instruments Can and Should Reduce Radiation Exposure

**DOI:** 10.1371/journal.pmed.1001884

**Published:** 2015-10-06

**Authors:** Emmanuel Lagarde

**Affiliations:** Institut National de la Santé et de la Recherche Médicale U897, Université de Bordeaux, Bordeaux, France

## Abstract

In this Perspective linked to Rodriguez and colleagues, Emmanuel Lagarde discusses the importance of decision instruments that can help physicians avoid subjecting patients to radiation exposure from unnecessary CT scans.

In this week’s issue of *PLOS Medicine*, Robert Rodriguez and colleagues publish the results of their validation study of procedures defined to decide whether chest computed tomography (CT) should be used for patients with blunt trauma [1]. The aim is to improve diagnosis while at the same time to identify patients for whom chest CT can be safely avoided. The procedures are meant to do so safely, with minimal risk of overlooking significant injuries that would have been seen using CT imaging. The decision instruments (DIs) selected by the authors included results from chest X-rays, the presence or absence of a rapid deceleration mechanism, and a set of five clinical observations. Analysis of patient data showed that about one out of three CT scans could be safely avoided using these DIs. The study by Rodriguez and colleagues is one of too small a number of papers attempting to identify solutions to the twofold concern of CT expansion: costs and exposure to ionizing radiation.

## Medical Ionizing Radiations Is Now a Concern

Ionizing radiation has long been known to increase cancer risk, evidenced in particular by the epidemiological follow-up of Hiroshima and Nagasaki bombings survivors, who were typically exposed to high radiation levels over a short period of time. A recent landmark study measured with unprecedented precision the association of long-term exposure to low-dose radiation with increased mortality from leukemia [2]. The authors estimated that each additional 100 mSv exposure raises the excess relative risk of leukemia death by 0.3. The yearly natural background radiation dose is 1–2 mSv, a chest X-ray is 0.01 mSv, and a chest CT radiation dose is about 7 mSv. Between 1998 and 2007 in the United States, the proportion of injury-related visits during which a CT was performed increased from 6% to 15% [3]. In developed countries, medically related radiation now accounts for more than half of the overall annual radiation dose received, the other half coming from radon in the atmosphere, cosmic rays, terrestrial radionuclides, and ingestion [4]. In addition to the necessity of reducing health system costs, we are therefore facing a new crisis with an urgent need to develop and validate alternative screening and diagnosis tools without compromising the patient’s prognosis. CT imaging is particularly on the spot, as it accounts for two-thirds of all medical radiation [5].

## Success Stories to Reduce Medical Radiation

The DIs described in the article by Rodriguez and colleagues join several previous examples of diagnostic algorithms that can reduce radiation exposure. Because of its very high (>99%) negative predictive value, serological measurement of S100-B protein shows potential to identify 20% to 30% of mild head trauma patients as having no need of imaging [6–7]. Traumatic brain injury, with an annual incidence of about two in 1,000, is the source of a huge proportion of CT-related ionizing doses, the highest (as measured in joule per kilogram of matter) when considering head CTs are focused entirely on one organ [8]. S100-B measurement is, however, not established as a systematic procedure in emergency departments, probably because its low specificity prevents it from fully replacing CT scan. Similarly, ultrasound detection of obstructive pyelolephritis is more and more often considered as an alternative to CT imaging [9]. Thoracic ultrasound in the diagnosis of pleural effusion, consolidation, pulmonary edema, and pneumothorax is also promising in this respect, but adequate validation studies are still missing [10].

## The Case of Children

The use of ionizing radiation in pediatric examinations is of particular concern for two reasons: children are probably more vulnerable to radiation (because of a higher rate of mitosis) and have a longer life span to develop long-term effects. Parsimony in the use of CT is therefore particularly important in children. A good example is the 50% substitution over 4 years of ultrasound for CT in appendicitis diagnosis in the US, with no adverse consequences [11]. This rapid change in practice was possible because ultrasound for appendicitis exhibits both relatively high (>90%) negative and positive predictive values (NPVs and PPVs), which has permitted both excluding appendicitis based on ultrasound examination (high NPV) and triage of children directly to surgery without need for CT imaging (high PPV). An inflammation-related biomarker panel has also been proposed to raise NPV close to 100% [12].

## Next Steps

One of the hurdles to CT use reduction is sometimes also the clinician’s reluctance to do without a powerful investigation tool that reduces the stress of uncertainty. The process of CT examination can, however, be stressful for patients; avoiding such anxiety—when safe to do so—might play a role in the recovery process. In addition to the data presented this week in *PLOS Medicine*, Rodriguez and colleagues also recently published the results of an original study showing patients’ increased willingness to discuss the pros and cons of CT prior to receiving imaging [13]. Facing the double challenge of high costs and evidence of cancer risk associated with accumulated low-dose radiation, research efforts should be promoted to identify new DIs based on biomarkers, clinical observation, and low or zero ionizing dose imaging tools and to evaluate those DIs that have already been proposed.

## Abbreviations

CT: computed tomography
DI: decision instrument
NPV: negative predictive value
PPV: positive predictive value

## References

[1] Rodriguez RM, Langdorf MI, Nishijima D, Baumann BM, Hendey GW, Medak AJ, et al Derivation and validation of two decision instruments for selective chest CT in blunt trauma: a multicenter prospective observational study (NEXUS Chest CT). PLoS Med. 2015; 12(10): e1001884. 26440607 10.1371/journal.pmed.1001883 PMC4595216

[2] Leuraud K, Richardson DB, Cardis E, Daniels RD, Gillies M, O'Hagan JA, et al Ionising radiation and risk of death from leukaemia and lymphoma in radiation-monitored workers (INWORKS): an international cohort study. Lancet Haematol. 2015 6; 2:e276–81. 10.1016/S2352-3026(15)00094-0 26436129 PMC4587986

[3] Korley FK, Pham JC, Kirsch TD. Use of advanced radiology during visits to US emergency departments for injury-related conditions, 1998–2007. JAMA. 2010 10 6;304(13):1465–71. 10.1001/jama.2010.1408 20924012 PMC11660594

[4] Abbott A. Researchers pin down risks of low-dose radiation. Nature. 2015 7 2;523(7558):17–8. 10.1038/523017a 26135428

[5] Mettler FA Jr., Wiest PW, Locken JA, Kelsey CA. CT scanning: patterns of use and dose. J Radiol Prot. 2000 12;20(4):353–9. 11140709 10.1088/0952-4746/20/4/301

[6] Biberthaler P, Linsenmeier U, Pfeifer KJ, Kroetz M, Mussack T, Kanz KG, et al Serum S-100B concentration provides additional information fot the indication of computed tomography in patients after minor head injury: a prospective multicenter study. Shock. 2006 5;25(5):446–53. 16680008 10.1097/01.shk.0000209534.61058.35

[7] Zongo D, Ribereau-Gayon R, Masson F, Laborey M, Contrand B, Salmi LR, et al S100-B protein as a screening tool for the early assessment of minor head injury. Ann Emerg Med. 2012 3;59(3):209–18. 10.1016/j.annemergmed.2011.07.027 21944878

[8] Wall BF, Haylock R, Jansen JTM, Hillier MC, Hart D, Shrimpton PC. Radiation risks from medical X-ray examinations as a function of the age and sex of the patient. HPA-CRCE-028. 66 pp. https://www.gov.uk/government/uploads/system/uploads/attachment_data/file/340147/HPA-CRCE-028_for_website.pdf

[9] Carnell J, Fischer J, Nagdev A. Ultrasound detection of obstructive pyelonephritis due to urolithiasis in the ED. Am J Emerg Med. 2011 9;29(7):843 e1–3. 10.1016/j.ajem.2010.07.006 20934827

[10] Ashton-Cleary DT. Is thoracic ultrasound a viable alternative to conventional imaging in the critical care setting? Br J Anaesth. 2013 8;111(2):152–60. 10.1093/bja/aet076 23585400

[11] Bachur RG, Levy JA, Callahan MJ, Rangel SJ, Monuteaux MC. Effect of Reduction in the Use of Computed Tomography on Clinical Outcomes of Appendicitis. JAMA Pediatr. 2015 8 1;169(8):755–60. 10.1001/jamapediatrics.2015.0479 26098076

[12] Huckins DS, Simon HK, Copeland K, Spiro DM, Gogain J, Wandell M. A novel biomarker panel to rule out acute appendicitis in pediatric patients with abdominal pain. Am J Emerg Med. 2013 9;31(9):1368–75. 10.1016/j.ajem.2013.06.016 23891596

[13] Rodriguez RM, Henderson TM, Ritchie AM, Langdorf MI, Raja AS, Silverman E, et al Patient preferences and acceptable risk for computed tomography in trauma. Injury. 2014 9;45(9):1345–9. 10.1016/j.injury.2014.03.011 24742979

